# Prognosis of patients with endometrial cancer or atypical endometrial hyperplasia after complete remission with fertility-sparing therapy

**DOI:** 10.1007/s00404-023-07077-7

**Published:** 2023-06-13

**Authors:** Hiromi Ga, Ayumi Taguchi, Harunori Honjoh, Akira Nishijima, Satoko Eguchi, Yuichiro Miyamoto, Kenbun Sone, Mayuyo Mori, Yutaka Osuga

**Affiliations:** 1 Department of Obstetrics and Gynecology, Graduate School of Medicine, The University of Tokyo, 7-3-1 Hongo Bunkyo-Ku, Tokyo, 113-8655 Japan; 2 Laboratory of Human Single Cell Immunology, World Premier International Immunology Frontier Research Center (WPI-IFReC), Osaka University, 3-1 Yamadaoka Suita-shi, Osaka, 565-0871 Japan

**Keywords:** Fertility-sparing therapy, Hysterectomy, Management after complete remission, Recurrence

## Abstract

**Purpose:**

Although many patients with endometrial cancer (EC) or atypical endometrial hyperplasia (AEH) achieve complete remission (CR) after high-dose medroxyprogesterone acetate (MPA) treatment, no consensus has been reached on management after CR. Currently, patients receive estrogen-progestin maintenance therapy, but no recommendations exist regarding the duration of maintenance therapy or whether hysterectomy should be considered. This study aimed to provide insights into the management of EC/AEH after achieving CR.

**Methods:**

We retrospectively investigated the prognosis of 50 patients with EC or AEH who achieved CR after MPA therapy. We assessed the association between disease recurrence and clinicopathological features and the pre- and post-operative histological diagnoses of patients who underwent hysterectomy.

**Results:**

The median follow-up duration was 34 months (range: 1–179 months). Recurrence was observed in 17 patients. Among the clinical characteristics investigated, only the primary disease was significantly associated with disease recurrence; patients with EC had a higher risk of recurrence than those with AEH (*p* = 0.037). During the observation period, 27 patients attempted pregnancy, and 14 pregnancies resulted in delivery. Patients who gave birth had significantly longer relapse-free survivals than those who did not (*p* = 0.031). Further, 16 patients underwent hysterectomies, and AEH was detected postoperatively in 4 of 11 patients (36.4%) with no preoperative abnormalities.

**Conclusions:**

We identified several clinical features of patients with EC and AEH after CR. Given the high probability of endometrial abnormalities detected postoperatively, hysterectomy may be considered for patients who no longer want children.

**Supplementary Information:**

The online version contains supplementary material available at 10.1007/s00404-023-07077-7.

## What does this study add to the clinical work?

**Table:** 

More than one-third of patients with EC/AEH after complete remission with high-dose medroxyprogesterone acetate treatment had undetectable endometrial abnormalities. Given the high probability of endometrial abnormalities, hysterectomy may be considered for patients who no longer want children.	

## Introduction

Medroxyprogesterone acetate (MPA) therapy is widely administered as a fertility-sparing therapy for stage IA, grade 1 endometrial cancer (EC) and its precursor, atypical endometrial hyperplasia (AEH). Generally, patients undergo total hysterectomy if MPA therapy fails to eliminate lesions. Although the response rate to MPA is as high as 53.2–79.9% [1–3], 9.5–40% of patients develop recurrence after complete remission (CR), and the median time to recurrence after CR is approximately 20 months [3–10]. However, despite the high recurrence rate of EC and AEH, management after achieving CR, such as whether to perform total hysterectomy after the patient gives birth, the timing of hysterectomy, and the duration of estrogen-progestin (EP) therapies, is likely to depend on the physician, institution, and patient.

Progestin is often used after complete remission, and cyclic low-dose progestin therapies or progestin-containing intrauterine devices are reported to lower the recurrence rate of EC and AEH [11]. In addition, previous reports have demonstrated that re-administration of fertility-sparing progestin therapy after recurrence has a good response rate in some patients [2, 3, 12]. Further, in patients with early EC, the CR rate for repeated therapy tends to be equal to or better than that of the initial treatment, and the pregnancy rate associated with repeated treatment is at least equivalent to that associated with the initial treatment [12]. Based on this evidence, a prospective phase II study to assess the efficacy of re-administration of MPA therapy for recurrent endometrial lesions after fertility-sparing therapy has been conducted in Japan. However, little evidence exists regarding the management of patients with EC or AEH after achieving CR with fertility-sparing therapies. In particular, there is little evidence on whether total hysterectomy should be recommended after giving birth and on the timing of hysterectomy. In this study, we retrospectively examined the prognosis of patients with EC or AEH after CR with MPA therapy, with a particular focus on the histological outcomes of patients who underwent total hysterectomy.

## Patients and methods

### Ethics approval and consent to participate

The protocol for this single-center, retrospective, observational study was implemented after being approved by the ethics committee of the Faculty of Medicine at the University of Tokyo (Approval number: 3084). The institutional review board granted an opt-out recruitment approach and waived the requirement for written informed consent from each patient. This study adhered to the principles of the Declaration of Helsinki.

### Patients and MPA therapy

This study included patients with EC or AEH treated with high-dose MPA therapy at the University of Tokyo Hospital between April 2006 and August 2022. Patients were administered 600 mg of MPA orally daily for 6 months, and if they did not achieve CR, MPA therapy was allowed to continue for up to 12 months. During MPA therapy, dilatation and curettage were repeated every 2 months to assess disease response. CR was defined as pathological CR confirmed using dilation and curettage. After CR, patients were usually administered oral EP until they wished to conceive. Endometrial biopsy was performed every 3–6 months, and when recurrence was suspected, patients underwent a more detailed pathological examination by dilatation and curettage, with or without hysteroscopy.

Patients were excluded if MPA therapy was started before April 2006, the therapy was not completed as of August 2022, CR was not achieved at the University of Tokyo Hospital, or follow-up data were lacking.

### Data collection and clinical outcomes

We obtained clinicopathological data, including age, body mass index (BMI), history of pregnancy and delivery, pathological diagnosis, and the International Federation of Gynecology and Obstetrics stage, from the electronic medical records. In addition, the durations of MPA and EP therapy were obtained, along with information on hysterectomies, operative procedures, and pathological diagnoses before and after hysterectomy. The cut-off value for BMI was 25 kg/m^2^. The duration of MPA therapy was categorized into two groups:  ≥ 7 months and < 7 months. “Recurrence of AEH or more” was defined as a pathological diagnosis of AEH or EC, and “recurrence of atypical glands or more” was defined as a pathological diagnosis of atypical glands, AEH, or EC.

### Statistical analysis

The Mann–Whitney *U* test was used to compare continuous variables, and the chi-square test was used to analyze categorical data. Fisher’s exact test was used instead of the chi-square test when the expected frequency of one or more cells was < 5. Relapse-free survival (RFS) was calculated from the date of the last MPA treatment to the date of the first diagnosis of “AEH or more” and “atypical glands or more” for “recurrence of AEH or more” and “recurrence of atypical glands or more,” respectively. The Kaplan–Meier method was applied to analyze RFS, and the log-rank test was used to analyze differences in survival based on various clinicopathological features. All tests were two-tailed, and *p* < 0.05 was considered statistically significant. All statistical analyses were performed using EZR statistical software [13].

## Results

### Patient characteristics and recurrence after CR

**Fig. 1:**
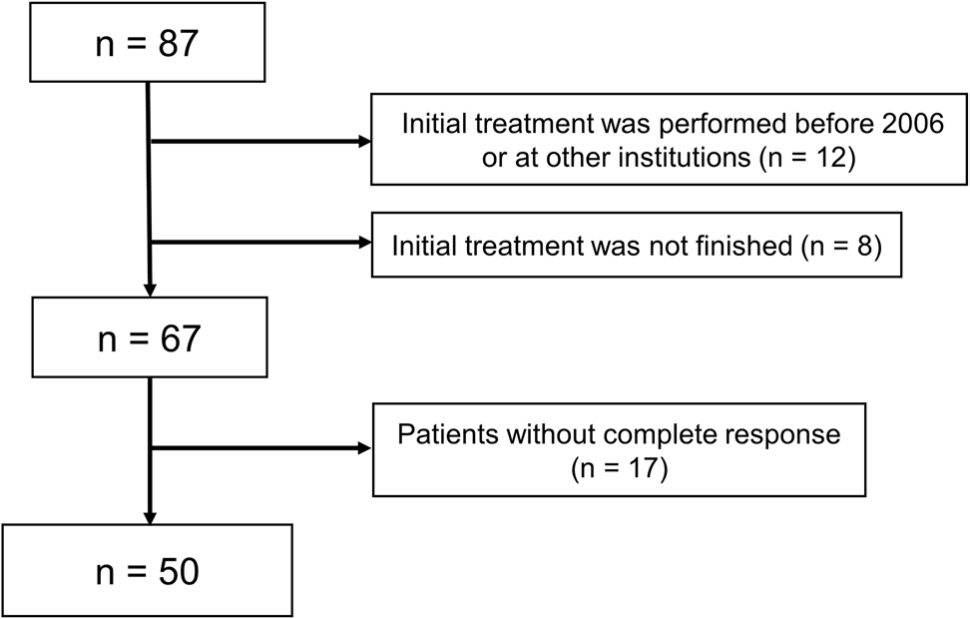
Flow chart of patient selection

Among the 87 patients treated with MPA in our hospital between 2006 and 2022, 50 achieved CR and were included in the study (Fig. 1). The clinicopathological characteristics of the 50 patients are summarized in Table 1. Of these, 19 patients (38%) had EC, and 31 (62%) had AEH. The median age and BMI were 32.6 years and 23.3 kg/m^2^, respectively. None of the patients had histories of pregnancy.

**Table 1:** Patients characteristics

Factors	Values
Age (years old), median, (IQR)	32.6 (29.3–36.8)
BMI (kg/m^2^), median, (IQR)	23.3 (19.4–25.7)
Parity, median, (range)	0 (0–0)
Primary disease, number (%)	
EC	19 (38)
AEH	31 (62)
MPA duration (months), median, (range)	6 (3–14)

*IQR* interquartile range; *BMI* body mass index; *EC* endometrial cancer; *AEH* atypical endometrial hyperplasia; *MPA* Medroxyprogesterone acetate

The median observation period from the end of MPA therapy was 34 months (range, 1–179 months); during this period, 16 patients (32%) had a recurrence of AEH or more, and 17 patients (34%) had a recurrence of atypical glands or more. The 2-year RFS rate was 72.3% (Fig. S1). Clinicopathological features of the patients with recurrence are summarized in Table 2. After recurrence, 11 patients (61.1%) were retreated with MPA; nine patients achieved CR, whereas two failed to achieve CR. Further, five patients underwent hysterectomy, and one patient with relapsed atypical glands was treated with oral EP and achieved CR (Table 2).

**Table 2:** Clinicopathological data of patients with recurrence

Case	Age at first recurrence	Number of delivery after CR	MPA duration (months)	Treatment after recurrence	Primary diseases	Recurrent diseases	RFS (months)
1	31	2	6	MPA	EC	EC	12
2	42	0	6	Hysterectomy	AEH	AEH	106
3	41	0	6	MPA	EC	AEH	21
4	45	0	6	Hysterectomy	EC	AEH	56
5	36	0	4	MPA	EC	AEH	23
6	44	0	6	Hysterectomy	EC	AEH	109
9	39	2	6	Hysterectomy	EC	AEH	68
11	41	1	6	MPA	EC	EC	21
13	35	0	9	MPA	AEH	AEH	13
14	35	0	6	MPA	AEH	AEH*	20
15	45	0	6	Hysterectomy	EC	AEH	50
16	38	0	6	MPA	AEH	AEH	10
26	39	0	6	MPA	AEH	AEH	9
27	35	0	3	MPA	AEH	AEH	16
28	35	0	11	EP	AEH	Atypical endometrium	3
29	31	0	6	MPA	EC	AEH	60
30	21	0	6	MPA	EC	AEH	21

*CR* complete remission; *MPA* Medroxyprogesterone acetate; *RFS* relapse-free survival; *EC* endometrial cancer; *AEH* atypical endometrial hyperplasia; *EP* estrogen-progestin

*The preoperative pathology was atypical glands, but the postoperative pathology was AEH

**Fig. 2:**
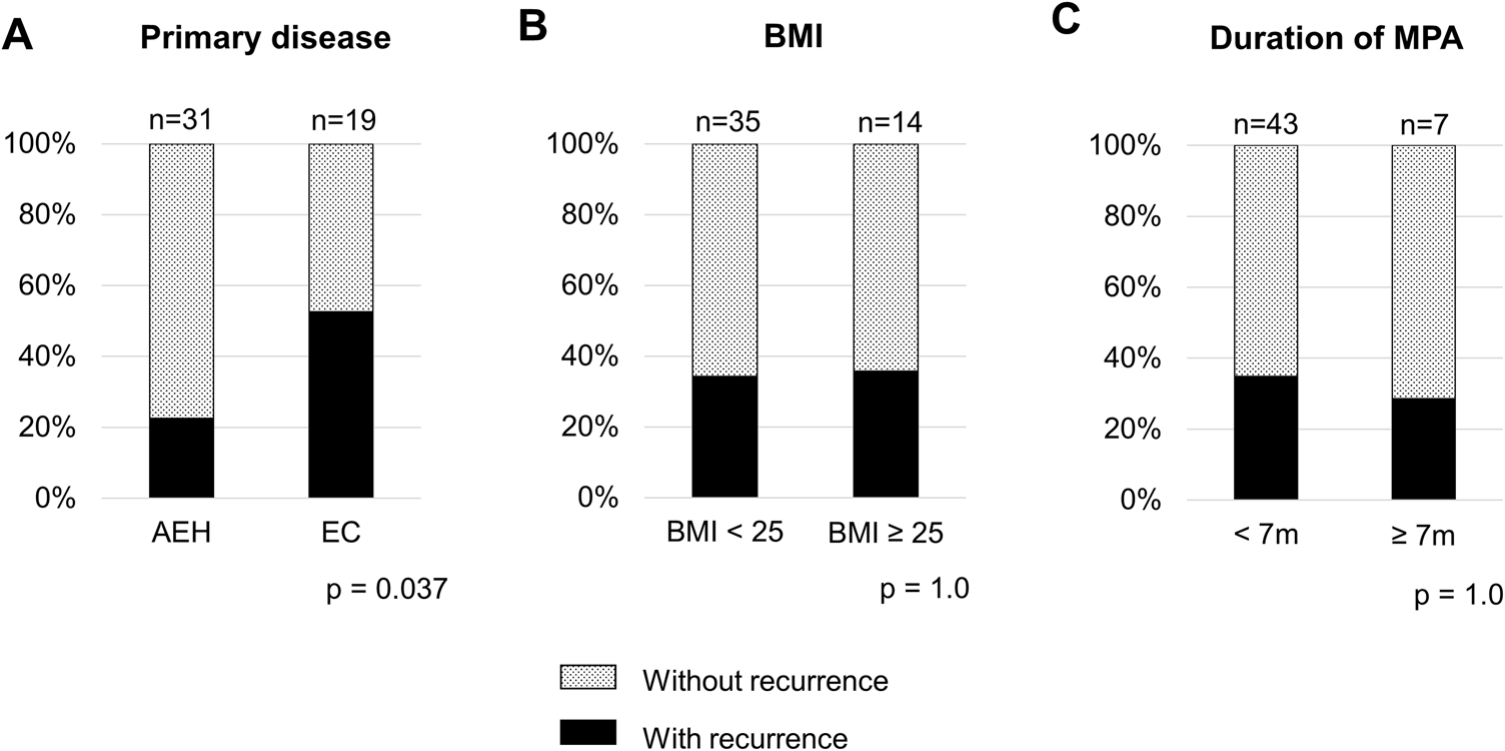
Association of clinical factors with disease recurrence. Association between disease recurrence and primary disease (**A**), body mass index (BMI) (**B**), and duration of medroxyprogesterone acetate (MPA) therapy (**C**). The chi-square test or Fisher’s exact test was used

The association between recurrence and clinicopathological characteristics was assessed. Only the primary disease was significantly associated with disease recurrence; patients with EC had a higher risk of recurrence than those with AEH (*p* = 0.037) (Fig. 2A–C).

### Pregnancy outcomes

**Fig. 3:**
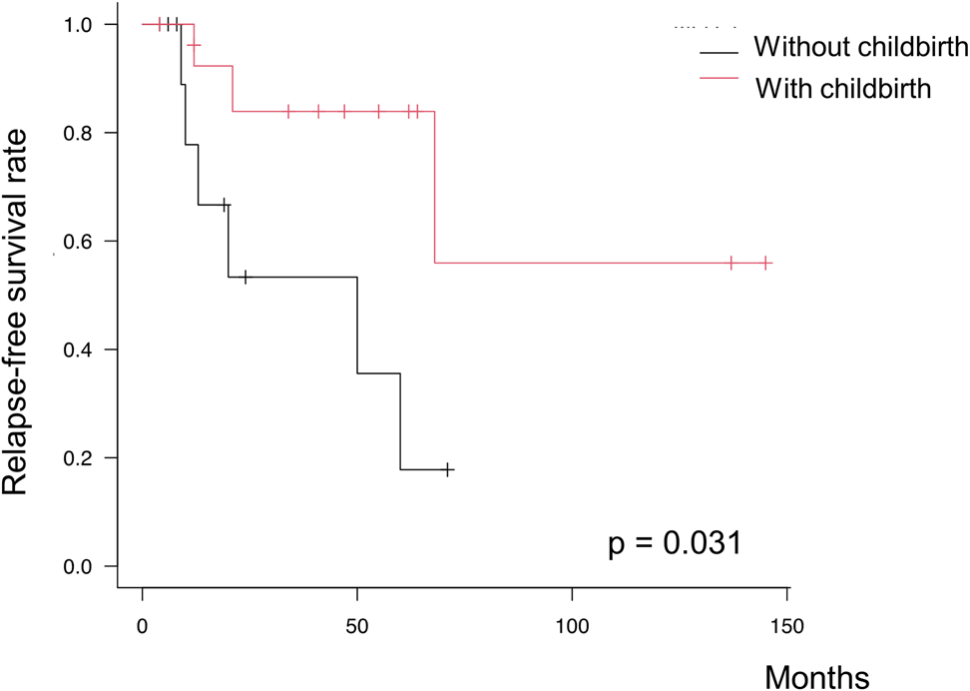
Relapse-free survival according to childbirth. Relapse-free survival rates according to childbirth among patients who tried to conceive. The log-rank test was used to assess differences in survival between groups

During the observation period, 27 patients attempted pregnancy, and 14 successfully delivered. Additionally, four patients underwent cesarean sections. Among the 27 patients who attempted pregnancy, those who gave birth were younger than those who did not (Table S1). When comparing RFS between patients who did and did not give birth, those who gave birth had a significantly longer RFS than those who did not (2-year RFS, 83.9% vs 53.3%; log-rank test, *p* = 0.0311) (Fig. 3).

### Surgical outcome

During the course of the study, 16 patients underwent hysterectomy. Among them, five patients underwent hysterectomy because of disease recurrence (1 with an atypical gland, 2 with AEH, and 2 with EC). The remaining patients decided to undergo hysterectomy based on a comprehensive consideration of other factors, such as age and desire to be pregnant. Table 3 shows summary of clinicopathological characteristics of patients who underwent hysterectomy after CR.The median age at surgery was 40.3 years (range, 31–49 years); six patients (37.5%) gave birth after initial MPA therapy. Additionally, seven patients continued EP maintenance therapy until they decided to undergo a hysterectomy.

**Table 3:** Summary of clinicopathological characteristics of patients who underwent hysterectomy after complete remission

Case	Age at surgery	Number of delivery	MPA duration (months)	Use of EP before surgery	Primary diseases	Preoperative pathology	Postoperative pathology
1	31	2	6	+	EC	Negative	AEH
2	42	0	6	−	AEH	Negative	AEH
3	41	0	6	+	EC	AEH	EC
4	45	0	6	+	EC	EC	EC
5	36	0	4	+	EC	Negative	Negative
6	44	0	6	+	EC	Atypical glands	AEH
7	49	0	10	+	EC	Negative	Negative
8	35	2	6	−	AEH	Negative	Negative
9	39	1	6	−	EC	Negative	AEH
10	40	1	5	−	AEH	Negative	Negative
11	41	1	6	−	EC	Negative	Negative
12	43	1	6	−	EC	Negative	Negative
13	35	2	9	+	AEH	AEH	AEH
15	45	0	6	−	EC	Negative	AEH
16	38	0	6	−	AEH	EC	EC
17	43	1	7	−	AEH	Negative	Negative

*MPA* Medroxyprogesterone acetate; *EP* estrogen-progestin; *EC* endometrial cancer; *AEH* atypical endometrial hyperplasia

It is noteworthy that AEH was postoperatively detected in 4 out of 11 patients (36.4%) with no preoperative abnormalities. In 3 of the 4 cases, EP agents were not used preoperatively.

## Discussion

In the current study, we retrospectively analyzed patients with EC/AEH who achieved CR with MPA therapy. We demonstrated that 17 of 50 patients (34%) developed recurrence, and 14 of 27 patients (51.9%) who attempted to conceive successfully gave birth. Further, 16 patients underwent hysterectomy, and 4 of 11 patients with no preoperative abnormalities were diagnosed with AEH postoperatively, indicating a high incidence of undetected residual or recurrent disease.

Of the 17 patients with recurrence, 11 relapsed within 2 years. Additionally, among patients who tried to conceive, RFS was significantly longer in patients who successfully gave birth than that in those who did not. These findings coincide with those reported in a previous study [11]. Two hypotheses surround the favorable prognosis of patients who give birth. First, pregnancy itself can reduce the recurrence rate of endometrial cancer because of the high levels of progesterone during pregnancy [14]. Additionally, the endometrium is shed during delivery, which is equivalent to curettage [3, 4]. The other hypothesis suggests that the molecular biology of the uterine endometrium that leads to endometrial cancer is similar to that of the uterine endometrium that makes implantation difficult. For example, loss of high-mobility group box-1 is associated with both endometrial cancer and implantation defects [15]. Considering the high recurrence rate within 2 years, patients who try but are unable to conceive after CR should be carefully followed up.

Notably, AEH was detected in the hysterectomy specimens of more than one-third of patients (four patients) without any preoperative abnormalities. As no cancer was missed, the clinical management was reasonable; however, it is important to consider that some neoplastic lesions may not be detected by endometrial biopsies. Additionally, 3 of the 4 patients did not have preoperative EP therapies. Although the association between EP therapy and complications of undetectable endometrial lesions is not clear due to the small number of cases, given a previous report that postoperative progestin administration prevents recurrence [11], patients who do not use EP agents should be followed more carefully. In addition, given the high residual rate of AEH detected postoperatively, hysterectomy may be considered for patients who no longer wish to become pregnant, regardless of the presence or absence of recurrence.

This study had some limitations. First, because of its retrospective nature, some data were missing. In addition, clinical management after CR is not uniform; thus, the duration of EP therapies varies from case to case. Second, this was a single-center study, and the sample size was relatively small. In particular, we were able to compare preoperative and postoperative pathology in only 16 patients. Further data accumulation is warranted to determine whether hysterectomy is recommended in patients in whom there is no evidence of recurrence after fertility-sparing therapies.

In conclusion, we confirmed that recurrence of EC or AEH, detectable by endometrial biopsy, is more common within 2 years after CR. Recurrence is more frequent in patients with EC than in those with AEH, and among patients who try to conceive, those who do not give birth have a higher risk of disease recurrence than those who do. In addition, we are the first to report residual or recurrent endometrial lesions after CR with fertility-sparing therapies, which are usually missed in follow-up. Our results indicate that the indications and timing of hysterectomy after CR may merit further examination.

### Supplementary Information

Below is the link to the electronic supplementary material.

Fig. S1 Relapse-free survival. Relapse-free survival rate of all patients who achieved complete remission after initial medroxyprogesterone acetate therapy. Supplementary file1 (PPTX 41 KB)

Supplementary file2 (DOCX 16 KB)

## Data Availability

The data that support the findings of this study are available from the corresponding author upon reasonable request after appropriate procedures.
