# Supplementary material for: Prognosis of patients with endometrial cancer or atypical endometrial hyperplasia after complete remission with fertility-sparing therapy
Source: Arch Gynecol Obstet. 2023 Jun 13;308(5):1629–34. doi: 10.1007/s00404-023-07077-7 (PMC10520125; doi:10.1007/s00404-023-07077-7)
Supplement: Supplementary file 1 — Relapse-free survival. Relapse-free survival rate of all patients who achieved complete remission after initial medroxyprogesterone acetate therapy. Supplementary file1 (PPTX 41 KB) [file 404_2023_7077_MOESM1_ESM.pptx]

## Slide 1
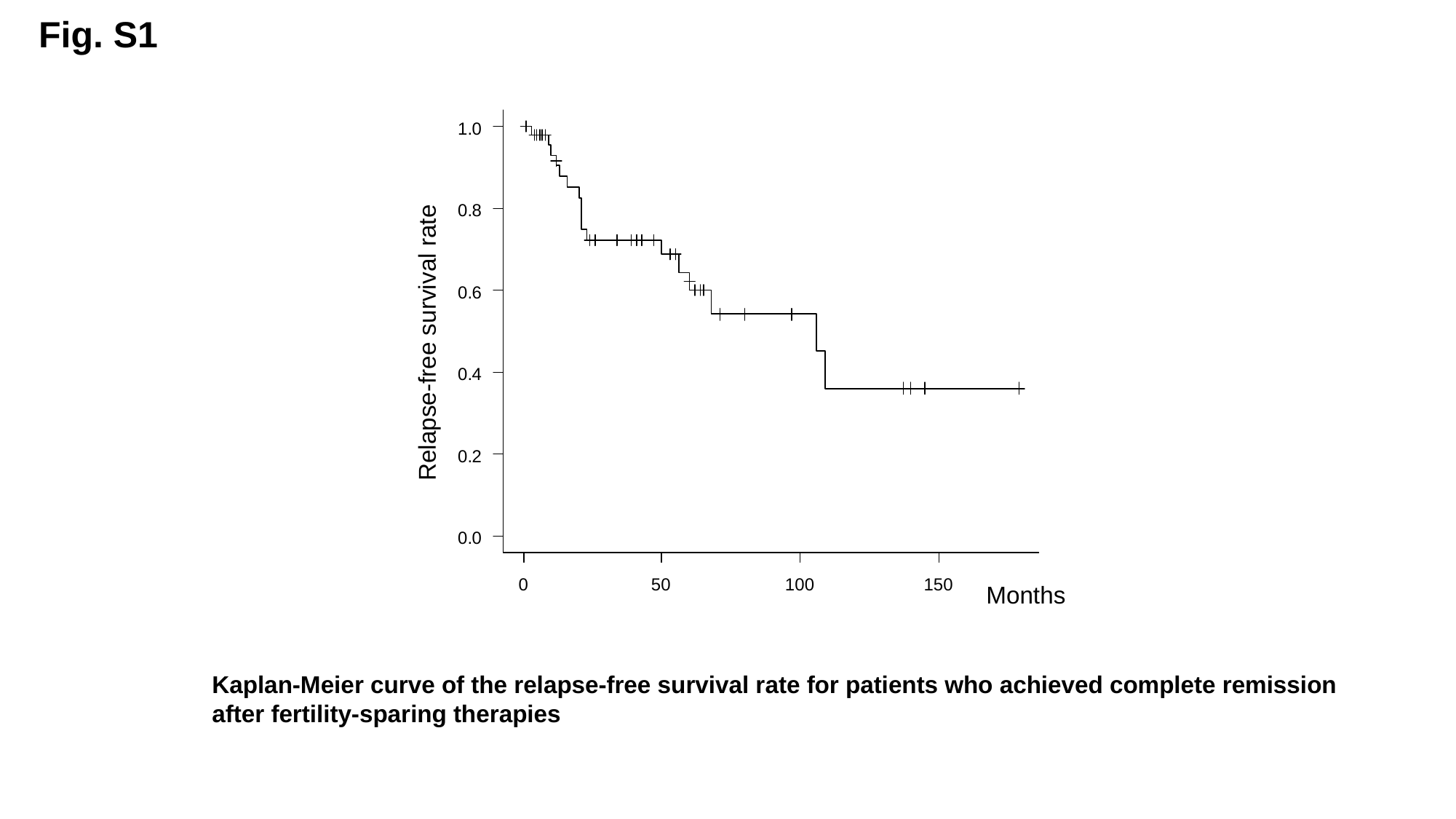

Fig. S1
Relapse-free survival rate
Months
Kaplan-Meier curve of the relapse-free survival rate for patients who achieved complete remission
after fertility-sparing therapies
