# Supplementary material for: Prognosis of patients with endometrial cancer or atypical endometrial hyperplasia after complete remission with fertility-sparing therapy
Source: Arch Gynecol Obstet. 2023 Jun 13;308(5):1629–34. doi: 10.1007/s00404-023-07077-7 (PMC10520125; doi:10.1007/s00404-023-07077-7)
Supplement: Supplementary file 2 — Supplementary file2 (DOCX 16 KB) [file 404_2023_7077_MOESM2_ESM.docx]

Table S1 Clinicopathological characteristics based on pregnancy outcome

|  | With pregnant  n = 14 | Without pregnant  n = 13 | p-values |
| --- | --- | --- | --- |
| Age at diagnosis, median, (IQR) | 31 (27.2-35.2) | 33 (31-36) | 0.163 |
| BMI (kg/m^2^),  median, (IQR) | 20.0 (18.9-21.1) | 20.7 (19.8-25.0) | 0.556 |
| Primary disease  EC, n (%)  AEH, n (%) | 8 (57)  6 (43) | 2 (15)  11 (85) | 0.0244 |

BMI, body mass index; EC, endometrioid carcinoma; AEH, atypical endometrial hyperplasia.
